# A road map to assess critical materials content in boron industrial wastes using sustainable micro-X-ray fluorescence and total reflection X-ray fluorescence instrumentation

**DOI:** 10.3906/kim-2003-33

**Published:** 2020-10-26

**Authors:** İbrahim KULA, Christian GUTSCHE, Yunus ERDOĞAN, Andreas FITTSCHEN, Ursula Elisabeth Adriane FITTSCHEN

**Affiliations:** 1 Department of Chemistry, Science Faculty, Muğla Sıtkı Koçman University, Muğla Turkey; 2 Institute of Inorganic and Analytical Chemistry, TU Clausthal, Clausthal-Zellerfeld Germany; 3 Department of Chemistry, Science and Art Faculty, Dumlupınar University, Kütahya Turkey

**Keywords:** Total reflection X-ray fluorescence, micro-X-ray fluorescence, boron, recycling

## Abstract

Turkey is the leading country in the world in terms of boron production and sale. Increasing boron production goes along with an increasing generation of boron wastes. The pollution of the soil and the air around the waste piles, as well as the occupation of several square kilometers of ground, are major environmental problems. It is, therefore, very important to make use of the wastes to both protect the environment and create revenue. This work presenteda road map for fast screening of boron waste for critical elements followed by determination of the elements using small footprint low power instrumentation. The sample preparation was kept to a minimum. A procedure that allowed an assessment of critical materials in industrial production waste with minimal consumption of hazardous acids, energy, and time was presented. The samples were first screened for valuable and hazardous elements by micro-X-ray fluorescence (XRF). Samples with considerable contents of Cs, Rb, and Aswere then prepared as slurries for the total reflection XRF (TXRF) measurement. To evaluate the TXRF procedure, a standard reference material was analyzed. As a result, Rb and Cs in concentrations up to 420 ± 70 and 1500 ± 200 mg/kg were detected in some of the waste forms. The time savings were in order of a factor of 3 when comparing the prescreening combined micro-XRF and TXRF approach to an all TXRFanalysis approach.

## 1. Introduction

Boron is an essential element in many modern technology materials. It is used in large amounts for the production offibers, specialty glasses, ceramic, enamels, detergents, metallurgy, and chemical manufacturing. Boron-based compounds are subject to research on advanced technology materials to be used in hydrogen storage [1], lithium-ion batteries [2], and superconductivity [3]. Boron production worldwide was 4.2 million tons in 2015, with 48% produced in Turkey. The most important commercial boron mineral colemanite (Ca2B6O11·5H _2_ O) reserves are found in Hisarcık, in the Espey regionof Turkey. Large quantities of boron wastes are discharged during the processing of the ore in concentrators. The amounts of colemanite solid wastes in Espey were 329,983tons in 2007 [4]. Millions of tons of waste were deposited around the ore plant over the years. It is, therefore, very important to make use of the wastes to both protect the environment and create increased revenue. As a consequence, many researchers have focused their efforts on the utilization of wastes stemming from boron mining and processing. Colemanite industrial waste has been used in many applications, such as additive material in ceramic and cement [5,6], absorbent to reduce contamination[7,8],and as a photo-catalyst [9]. To assess the amount of critical materials, it is necessary to subject those to a trace elemental determination procedure. Colemanite waste has been evaluated previously for Li and Rb using an approach involving inductively coupled plasma optical emission spectrometry (ICP-OES) [10–12]. However, this procedure requires the complete dissolution of the respective waste sample, usually involving high concentrated acids like HF and microwave digestion. Herein, an alternative potentially more sustainable procedure using low-power (50W) X-ray fluorescence (XRF)equipment was presented. First micro-XRF was used to screen different wastes forcritical elements like Cs and Rb and hazardous elements like As. Subsequently, total reflection XRF (TXRF) analysis was used for elemental determination. Neither procedures required sample digestion. The samples were prepared as slurries and were not consumed during the measurement, which was an advantage if follow-up analysis or treatments were required.

In micro-XRF, an X-ray optic is used to restrict the excitation beam size or focus the excitation beam to a small spot (10–50 µm) on the sample so that small features on the sample can be analyzed [13].This allows for elemental mapping of a sample through the beam as well as for screening several samples in a row. This technique has been widely used to analyze environmental samples [14–16], cultural heritage materials [17,18], and for nuclear material characterization [19,20]. In the study presented herein, micro-XRF was used to screen spot-likedeposits of different colomanite mining waste. This allowed for a rapid screening of waste fractions that were potentially valuable for recycling. Additionally, it was used to map the elemental distribution in the TXRF slurry specimens to identify potential bias arising from within homogeneities.

After the micro-XRF screening, selected samples with high concentrations of Cs and Rb were subjected to slurry-sampling TXRF. Cs and Rb are of commercial interest, as the demand for these elements has been constantly growing for 2 decades, and their prices have increased in the global market [21]. In addition to other uses, Rb is used in specialty glasses for manufacturing fiber optics, night-vision devices, and photomultiplier tubes [22]. The largest present-day use of nonradioactive Cs is in cesium formate drilling fluids for the extractive oil industry. Rb and Cs display very similar electrical, chemical, and physical properties, and consequently, their detection techniques are very similar.

TXRF is an energy-dispersive XRF technique providing simultaneous multielement detection. Total reflection is achieved by impinging the primary beam on the sample carriers at an angle below the critical angle of total reflection. This way, the background is reduced tremendously when compared to conventional XRF geometries. This allows one to determine most of the elements with detection limits in the ppb and sub-ppb range comparable to ICP-OES. In TXRF, only minute sample amounts in the sub-mg range are needed, as modern instruments run with low power X-ray tubes (50W) and electrically cooled semiconductor detectors. Additionally, TXRF is nondestructive [23].The analysis of refractory material is generally enabled by slurry sampling with minimal use of chemicals. TXRF has been used previously to study the effectiveness of recycling valuable materials from copper-smelter [24].It has also shown its potential for different applications including environmental [25], nanotechnology [26], geological materials [27], and forensic sciences [28]. Nonetheless, the minimal sample preparation also poses challenges with respect to homogeneity of the specimen, as the sample might consist of particles differing in size and composition. Sedimentation may take place during sample preparation and alter the measured overallstoichiometry. Precisionandaccuracyofslurry-samplinginTXRFismainly influenced by particle size distribution and homogeneity of the internal standard (ISTD) and the analyte in the specimen. Powders consisting of unimodal small particles are ideal for the analysis, but often not available without significant pretreatment. Therefore, surfactants and stabilizers are used to increase the density of the suspension and prevent particles from agglomerating. In the current approach a grinding step and the addition of a detergent, namely Triton-X-100, was applied. The accuracy of the procedure was evaluated using certified reference material (CRM).

## 2. Experimental

### 2.1. Chemicals and samples

Deionized water (ELGA-VEOLIA, England) with a minimum resistivity of 18.2 MΩ ∗cm at 25 °C) and analytical grade reagents were used for all preparations of the standard and sample solutions. Triton X-100 (DOW, Midland, USA) solution was used to stabilize the suspensions.

An ISTD of 1000 mgL ^-1^ of Ga (Merck KGaA, Darmstadt, Germany) was used for calibration purposes. A silicone solution in isopropanol (SERVA Electrophoresis GmbH, Heidelberg, Germany) was used to cover the quartz carriers and make the surface hydrophobic. The CRM WQB-1 lake sediment from the National Water Institute of Canada was used to evaluate the analytical procedure.

Most of the boron industrial wastes were obtained from the Emet Etibor Factory in the Espey and Hisarcık regions of Turkey. This region is within the borders of Kütahya Province, which is located in inner west Anatolia. At the Espey site,samples F02 (0:zone colemanite), F09 (2: zone colemanite clay), F10 (3: zone colemanite clay), F13 (lower 25 mm of diameter solid waste), F16 (25–100-mm of concentrated colemanite), F18 (0–3-mm diameter of concentrated ore), F21 (lower 10-mm diameter of stock sample), and F25 (dam waste, muddy) were collected. At Hisarcık, samples FH8 (3–25-mm diameter of concentrated colemanite) and FH20 (bottom colemanite ore) were taken. Enriched aluminum based (alunite) powder (A1) was obtained from Şaphane and Tincal raw material (Na2O.B2O3.10 H2O) from Kırka.

### 2.2. Instrumentation and sample preparation

#### 2.2.1. Micro-XRF

A micro-XRF unit built in-house, which was described in detail in [29] was used to screen an aliquot of each waste sample. The micro-XRF unit consisted of a 50-W micro focus Rh-tube with an polycapillary optic (XOS, Albany, NY, USA) attached and an energy-dispersive silicon drift detector (Vortex, Hitachi High-Technologies Science America, Inc., Chatsworth, CA, USA). The primary beam was focused to a diameter of about 50 µm. The samples were prepared on a 4-µm thick polypropylene foil (Ultralene, SPEX, Metuchen, USA). For the screening procedure, approximately 1 mg of each sample was prepared equidistantly on the foil. An optical microscope component of the micro-XRF setup was used to navigate between the specimens. Spectra were acquired using a live time of 100 s for each deposit. The spectra were deconvoluted using PyMCA software [30]. Because of the partly unknown low Z matrix, the fluorescence lines of a series were mostly fitted separately. For the study of the homogeneity of the dried deposit, 10 µL of the slurry obtained from sample F13 was prepared on an Ultralene foil, and scanned with the focused beam with a step size of 10 µm and a live time of 1 s. The preparation of the slurries is described in the following paragraph.

#### 2.2.2. TXRF

A TXRF spectrometer (S2 PICOFOX; BrukerAXSnano, Madison, USA)equipped with a molybdenum (Mo) air-cooled X-ray tube (600 µA, 50 kV, 50 W) and a Peltier-cooled XFlash silicon drift detector with 30 mm ^2^ active area [detector resolution >150 eV at 100 kcps (MnKα)] was used to determine elements in the boron wastes and the CRM. A curved multilayer monochromater was used to select the Mo Kα (17.4keV) from the tube spectrum. The beam then impinges on the sample support-here a quartz reflector- with an angle of approximately 0.05°, which is less than the critical angle. Prior to the TXRF measurements the samples were ground for 15 min with an agar mortar. A sample amount of 5 mg was suspended in 2.5 mL of a 1% Triton-X solution. As an ISTD, 10 µL of a Ga solution with a concentration of 99.8 mg/L was added to 990µL of the suspension to give a final volume of 1000 µL. The suspensions were sonicated in an ultrasonic bath for 15 min, and finally, vortexed just before preparation. By using the grinding+ultrasonic bath+vortex procedure, the particles stayed dispersed in the solution with no visible signs of precipitation. From this mixture, 10 µL were prepared on quartz reflectors for the TXRF analysis. The absolute amount of Ga on the reflectors was 9.98 ng Ga, and that of boron waste and SRM sample was 19.8 µg. TXRF measurements were performed with 300, 900, and 1000 s live time. However, increasing the analyzing time resulted in no notable decrease of the relative standard deviation; therefore, a measurement time of 300 s was employed for all of the samples. The number of replicates was at least 3 for all of the experiments.

#### 2.2.3. Particle sizer

For assessing the particle size distribution of the samples, a Mastersizer S (Malvern Instruments, Houston, TX, USA) was used. This instrument enables the determination of the particle size and size distribution of solid particles in the size range between 50 nm and 900 µm based on dynamic light scattering. From each sample, 2.0 g were mixed with 25 mL of sodiumhexametaphosphate solution (5 g/L) and stirred for 3 days. The particles were than dispersed into the liquid that circulated across a quartz measurement cell illuminated by a laser beam. The fitting of the curves and statistical evaluation were performed with MATLAB (R2018a, MathWorks, Inc., Natick, MA USA).

## 3. Results and discussion

### 3.1. Particle size distributions

**Figure 1:**
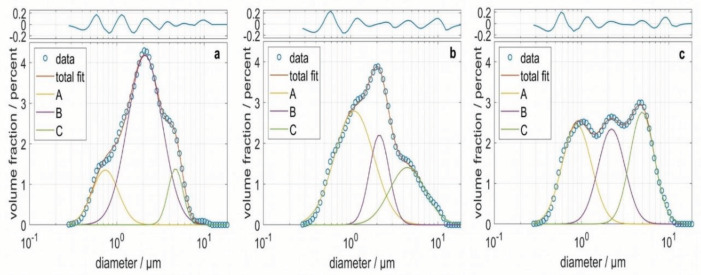
Particle size distribution of a CRM (a) and 2 samples, F13 (b) and F21 (c). Experimental data are plotted with circles.The totalbfit based on 3 log-normal distributions as well as the 3 partial particle size distributions, A, B, and C, are shown as solid lines. The residuebis shown on top.

The particle size distribution of the CRM and 2 exemplary samples was determined via dynamic light scattering. The particle size distribution was fitted using a small, medium-sized, and large particle fractions, A, B, and C, which were log-normal distributed, respectively. The experimental data, as well as the fits and the residue, are shown in Figure 1. The goodness of the fit was satisfying. The mean diameters, standard deviations, and volume fractions of the 3 particle fractions in each sample, as well as the coefficient of determination R ^2^ , are shown in Table 1. The mean values for the 3 fractions were in the range between 0.7 and 4.9 µm and differed by about less than 1 order of magnitude.

**Table 1:** Fit parameters and residue for the samples CRM, F13, and F21.

	CRM	F13	F21
Volume fraction of A/%	6	28	12
Mean diameter of A/µm	0.7	1.1	0.9
Standard deviation of A/µm	1.4	1.7	1.5
Volume fraction of B/%	73	20	26
Mean diameter of B/µm	2.1	2.1	2.2
Standard deviation of B/µm	1.6	1.3	1.4
Volume fraction of C/%	21	52	62
Mean diameter of C/µm	4.7	4.5	4.9
Standard deviation of C/µm	1.2	1.6	1.4
R ^2^	0.9973	0.9955	0.9958

### 3.2. Micro-XRF screening

**Figure 2:**
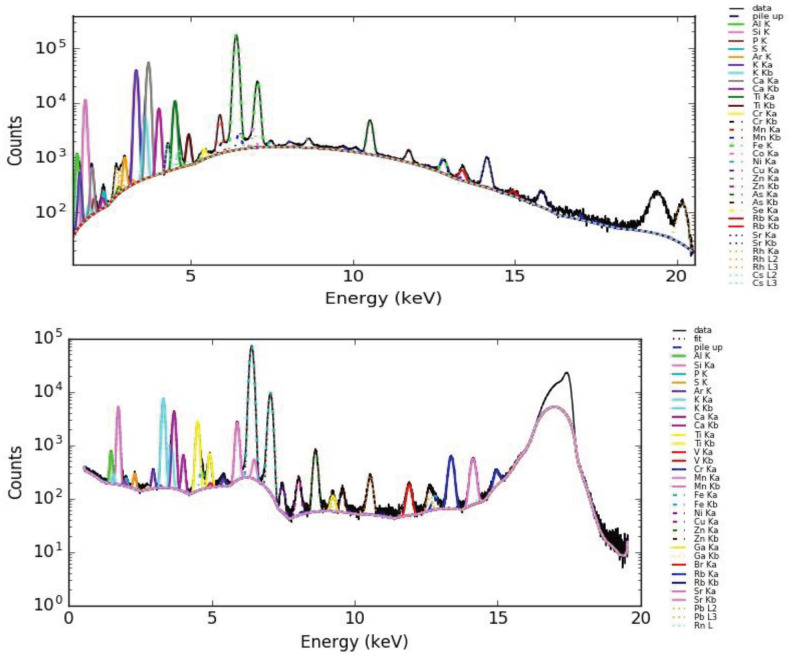
Fitted spectrum of sample F13 obtained by micro-XRF (a) and fitted spectrum of the CRM obtained by TXRF (b).

Twelve samples from different waste forms originating from boron ore processing were screened for valuable elements, such as Rb and Cs, and hazardous elements, such as Pb and As, using micro-XRF as described in the experimental part. Mass ratios were determined from these spectra using sensitivities determined previously [29]. The contents of major elements, as well as potentially valuable and harmful elements, are shown in Table 2, wherein relatively high amounts of potentially valuable and hazardous elements are underlined. A fitted spectrum of sample F13 obtained by micro-XRF is shown in Figure 2a. Note that light elements Z < Al were not accounted for in the screening step. Waste samples A1 and A7 contained considerable amounts of Ga and Ge. They also contained the highest amounts of As and Pb. Samples F13 and F21 had relative high amounts of Cs or/and Rb. Sample F25 also contained Cs and Rb; however, they originated from a source that was not interesting from a recycling perspective. In consequence, samples F13andF21 from Colemanite waste from the Espey region of Turkey were selected for further investigation with TXRF. The 2 selected samples also showed relatively high concentrations of As and Pb.

**Table 2:** Relative elemental amounts in % derived from micro-XRF screening. The amounts are normalized to the total amounts of elements detectable with micro-XRF.

Element	F09	F10	F13	F16	F18	F25	A1	A7	F2	F21	FH8	FH20
S	0.1	0.2	0.1	0.02	0.04	0.2	18	0.5	0.2	0.1	0.2	1.8
K	0.9	3	18	2.5	4	13.0	49	2	2	6	1	2
Ca	94	91	19	90	78	31	1	44	81	46	76	70
Fe	1	3	41	4	8	43	7	7	9	22	3	2
Cu	0.4	0.1	0.4	0.2	0.2	0.2	0.9	1.3	0.2	0.8	0.5	0.1
Zn	0.3	0.2	0.7	0.2	0.4	0.5	0.8	1.3	0.3	1.0	0.6	0.2
Ga	0.1	0.1	0.3	0.1	0.1	0.2	1.3	0.8	0.2	0.5	0.7	0.0
Ge	0.1	0.05	0.1	0.1	0.2	0.1	0.3	0.6	0.2	0.3	0.7	0.1
As	0.2	2	7	0.1	0.3	1.3	1.5	20	0.2	7	0.2	16
Se	0.2	0.1	0.2	0.2	0.6	0.1	0.7	0.6	0.2	0.5	0.6	0.2
Rb	0.2	0.1	0.8	0.2	0.6	1	0.4	0.3	0.3	1.3	0.7	0.1
Cs	0.03	0.02	0.8	0.05	0.2	0.8	0.01	0.05	0.1	0.3	0.2	0.0
Pb	2	0.5	8	2	6	4	14	6	3	7	5	5

### 3.3. TXRF investigation

Prior to the TXRF analysis of the selected samples, a CRM was analyzed to evaluate the analytical procedure. The CRM lake sediment CRM (WQB-1, National Water Institute Canada) was subjected to the same preparation procedure as the samples. The results from the elemental determination, as well as the certified results and the recovery, are shown in Table 3. Italic values differ more than 20% from those given in the certificate. Ga was used as the ISTD, because Ga was also present in minor amounts in the CRM and the ISTD concentration was corrected for this amount in Table 3. A fitted spectrum of the CRM obtained by TXRF is shown in Figure 2b. The lower background obtained in the TXRF measurement is due to the monochromatic excitation and the small excited volume. Only, the microscopic sample and the surface most 1–5 nm of the sample support were irradiated using the total reflection geometry.

**Table 3:** Elemental amount in lake sediment CRM (WQB-1, National Water Institute Canada) determined with TXRF, the certified amounts and the recovery are displayed.

Element	Concentration(µg/g)	Limit of detection(µg/g)	Certifiedconcentration (µg/g)	Recovery %
K*	25395	29	30496*	83
Ca*	9252	19	11672*	79
Ti*	3561	12	2862*	124
V	73	9	129	57
Cr*	168	7	89.1*	189
Mn	2107	6	2237	94
Fe	44959	4	47358	95
Ni	48	2	61.5	78
Cu	72	2	79.6	91
Zn*	260	2	275*	95
Rb	134	1	152	88
Sr*	133	2	146*	91
Pb	91	2	83.7	109
Ba	1368	31	606	226

* Uncertified value.

Overall, the TXRF analysis determined lower amounts than certified. This can be explained by a loss of sample material due to the sedimentation of usually larger particles. The sedimentation can also be influenced by the density fluctuations within the sample due to compositional variations, or in other words, particles with a higher density tend to sediment faster. Moreover, the polarity of the particle surface might play a role that depends on the particle composition. Therefore, these dimentation can, in addition to a changes in the size distribution, also lead to changes in the sample composition. Considering this loss of material and assuming no compositional changes, one would expect recovery rates of 91%. The deviations of the recovery rates for Ba, Cr, and V might be explained by the inhomogeneity of the sample and thus, inhomogeneous losses during the preparation.

**Figure 3:**
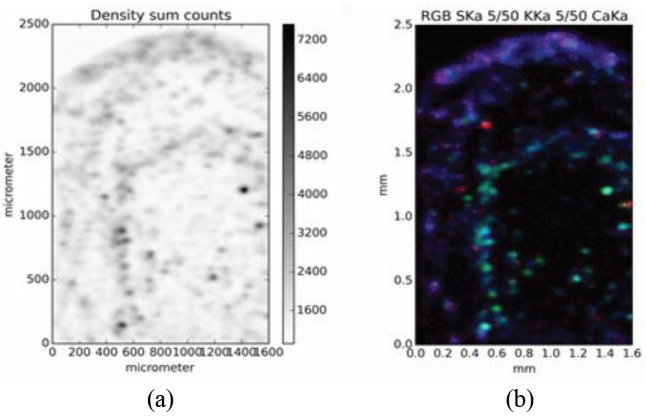
Image displaying the sum over their entire spectrum of each pixel is shown in (a) and a false color image showing the distribution of S (red), K (green), and Ca (blue) is shown in (b).

As mentioned above, uncertainties in the TXRF analysis of the slurry samples may arise from inhomogeneous specimens. Usually, an ISTD calibration is used for elemental quantification. The concept of internal standardization in TXRF is valid if the analyte and the standard element are homogeneously distributed. Herein, the homogeneity was evaluated by micro-XRF. To limit the scanning time, a fraction of the slurry deposited on a 4-µm-thick ultralene film was scanned with the micro-XRF. In Figure 3a, a density image of the sample is shown, depicting the sum intensity of the whole spectra. Exemplary elemental maps of S (red), K (green), and Ca (blue) show that the composition of the particles was not uniform (Figure 3b). It indicates that Ca was enriched at the rim of the dried deposit.

The data can be statistically evaluated concerning correlations. By that, one can track elements that are not easily detectable but correlated with elements showing more intense signals. The statistical evaluation of the data showed a strong correlation between K and Fe, indicating that those elements occurred together in a compound.The Pearson correlation coefficient of K and Fe was 0.92. The correlation coefficient of the ISTD Ga and Rb and between Ga and Cs was found to be 0.27 and 0.39, respectively. This means that there was no significant correlation between the 3 elements. However, the microhomogeneity (approximately10µm ^2^ ), which was shown by the pixel-by-pixel correlation of the elements, was not a good indicator of inhomogeneities relevant for TXRF. In TXRF, bias mainly occurs because the specimen is not entirely detected, accordingly, the macroscopic inhomogeneity on the length scale of 0.1 mm ^2^ is more relevant. For example, the rim of a specimen is not covered by the detector solid angle. To evaluate the magnitude of large area inhomogeneities, the mapped area was divided into 5 sections. Elemental concentrations were determined for each individual section using Ga as the ISTD and then the deviations between those were determined. The RSTD for Rb was 1.3%. A 2% deviation for As and 1.3% deviation for Cs were found. However, for the higher concentrated Fe, a RSTD of 14% was found, which can be explained by a higher degree of inhomogeneities. Accordingly, if the specimens were probed only in part by the TXRF setup, a deviation in this magnitude may have occurred.

### 3.4. Waste analysis

**Figure 4:**
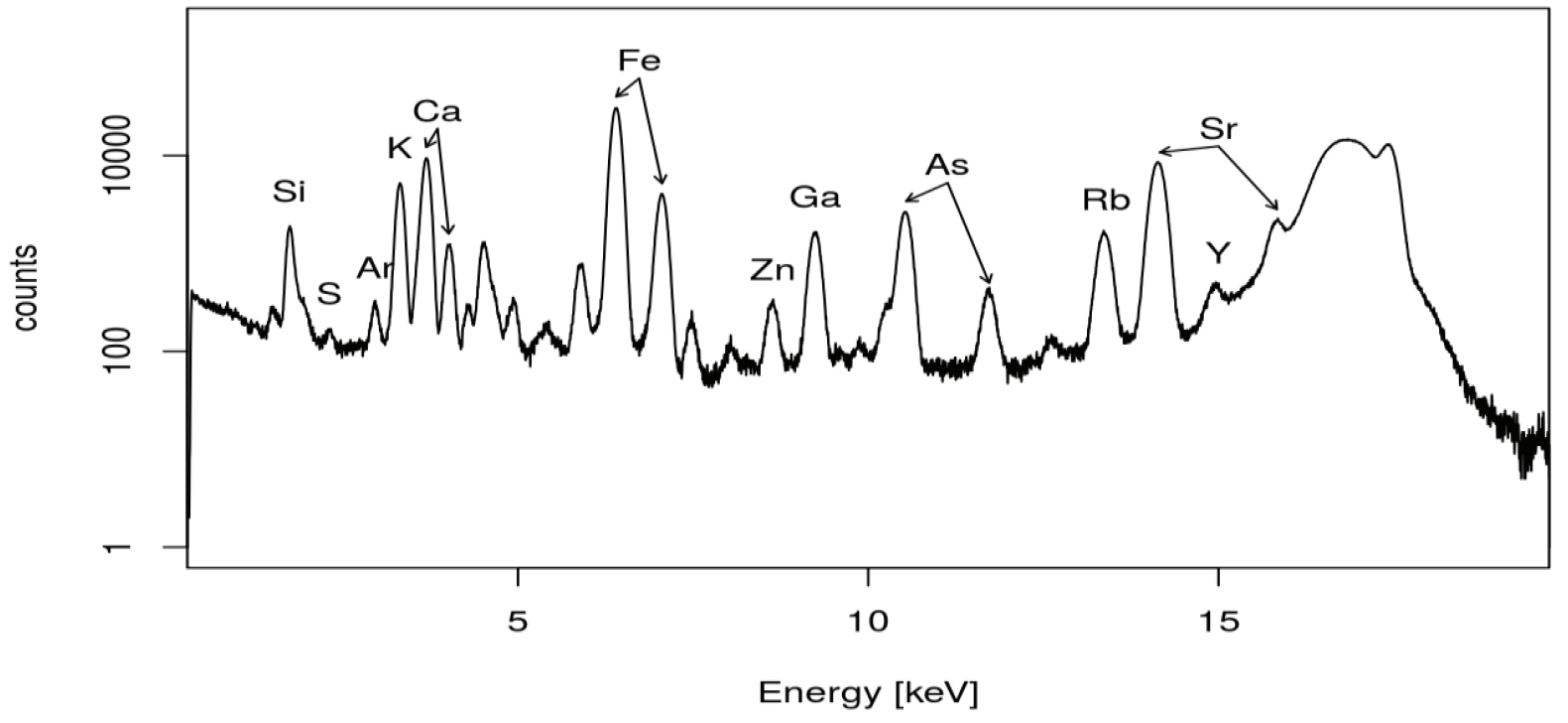
TXRF spectrum of the boron waste (F13) with Ga as the ISTD.

With this knowledge, the 2 samples from Colemanite waste from the Espey region of Turkey, F13 and F21, and in addition, the crystalline boric acid and boric acid powder from the boric acid plant, and an Alunite sample (KAl _3_ (SO _4_ ) _2_ (OH)6) were analyzed. The results obtained from the TXRF measurements are given in Table 4. Potentially hazardous and valuable elements are underlined in the Table. A TXRF spectrum of the boron waste sample (F13) is shown in Figure 4.

**Table 4:** Concentrations of the elements found in the different samples (mg/kg) by TXRF measurements N = 3, and the RSTD in % is given in brackets.

	F13	F21	Boric acid(crystalline)		Alunite (powder)
K	14000 ± 2000 (14)	9000 ± 300 (3)	-	130 ± 9 (7)	163000 ± 3000 (0.2)
Ca	14000 ± 1000 (7)	68000 ± 2500 (4)	1010 ± 50 (5)	70 ± 5 (7)	9800 ± 300 (3)
Sc	-	-	-	-	220 ± 30 (14)
Mn	600 ± 100 (17)	180 ± 7 (4)	-	-	720 ± 40 (5)
Fe	16000 ± 2500 (16)	6900 ± 270 (4)	40 ± 9 (20)	16 ± 1 (6)	21900 ± 400 (2)
Ni	50 ± 7 (14)	20 ± 2 (10)	-	-	425 ± 25 (6)
Cu	19 ± 1 (5)	9 ± 2 (22)	70 ± 6 (9)	9 ± 1 (11)	810 ± 30 (4)
Zn	100 ± 20 (20)	40 ± 2 (2)	7400 ± 35 (0.5)	11 ± 1 (9)	1990 ± 50 (3)
Ga	-	-	-	-	290 ± 30 (10)
Ge	15 ± 3 (20)	-	-	-	-
As	1000 ± 100 (10)	1600 ± 200 (13)	-	-	700 ± 25 (4)
Br	-	220 ± 6 (3)	400 ± 7 (2)	-	-
Rb	420 ± 70 (17)	130 ± 15 (12)	-	-	80 ± 10 (13)
Sr	2000 ± 40 (2)	3200 ± 100 (3)	-	-	2800 ± 60 (2)
Cs	1500 ± 200 (13)	330 ± 40 (12)	-	-	-
Pb	870 ± 50 (6)	7 ± 1 (14)	-	-	90 ± 20 (20)

For calibration, Ga was added as the ISTD. Because the samples already contained minor amounts of Ga (see micro-XRF results), the samples were analyzed by TXRF prior to the addition of Ga. The relative amount of Ga was determined. Finally, the applied ISTD concentration was adjusted by this value.

With respect to the recycling aspect, the concentrations of Rb and Cs in samples F13 and F21 were reasonably high; thus, recovery of these elements can be attractive. However, the Pb concentration in sample F13 was fairly high,at 870 mg/Kg, as well as the As concentration in both sample F13 and F21,at 1000 and 1600 mg/Kg, respectively. Eventually, the efficiency to separate those into a disposable form of lower volume will also be important to assess the recycling strategy. When compared to the micro-XRF screening, the relative amounts deviated from those obtained by the TXRF analysis. The deviation between the micro-XRF and TXRF results were found to be between factors of 1 to 4 for most elements. For example, in sample F13, the K and Ca relative contents were underestimated by a factor of 2 by the micro-XRF. Cs concentrations were 3 times higher than expected from the micro-XRF screen of sample F21. On the other hand, the Rb concentration was matched fairly well. This was due to the relatively high uncertainty of the micro-XRF analysis. The relative amounts given in Table 2 refer to the total of all of the detectable elements with a cut-off to the low energy side at about 2 keV. Major elements like O _2_ , Si, Al, and C were not accounted for in the normalization. On the other hand, in the TXRF data processing, all of the concentrations were derived from the normalization to the prepared sample masses. Additionally, in the micro-XRF, the values were altered via matrix effects. These effects were considered to be nonexistent in the TXRF measurements. The optimization of the semiquantitative approach of the micro-XRF, by accounting for the light elemental matrix, will further improve the accuracy of the screening. The time saved by screening naturally depends on the relation of the screened to eventually fully analyzed samples. Herein, 2 of 12 the samples were selected. This reduced the pure measurement time by half. The preparation time of the TXRF samples was also longer than that for the micro-XRF screening. In both cases, the samples needed to be prepared onto their respective sample carriers (1 min/sample). The TXRF determination, however, also requires finer powders (grinding 10 min/sample), and to weigh an aliquot of the sample (1 min/sample), the slurry with the ISTD needs to be prepared and the specimens need to be dried. However, herein, several specimens may have dried together, so there was no gain if 2 or 12 samples were prepared. Overall, the waste analysis time in this example was reduced by a factor of 3 (approximately 1 h with micro-XRF screening versus approximately 3 h when all of the samples were analyzed by TXRF). The actual savings in preparation time, however, will also depend on the state of automation of the sample preparation procedure of the individual laboratory.

## 4. Conclusion

The micro-XRF screening allowed to for rapid screening of the selected samples and gave a good estimate of the concentrations later determined by TXRF. The micro-XRF screening also revealed that elements of potential interestfor recycling, other than Cs and Rb, such asGa, were present in some of the waste forms. This highlighted the nontarget screening capacities of the TXRF approach. In this study the prescreening reduced the analysis time by a factor of approximately 3. In the TXRF analysis, the concentration of Cs was found to be as high as 1500 ± 200 mg/kg and Rb was as high as 420 ± 70 mg/kg in some waste forms. The slurry sampling TXRF analysis had the advantage of completely avoiding the consumption of hazardous acid and ultra-pure argon, which are necessary when using ICP-OES or ICP-MS. The study of an CRM showed a recovery of approximately 90% for most of the elements. This may have been attributed to a potential loss of large particles. The distribution of the ISTD and the analytes was found to be fairly homogeneous for the elements of interest. In summary, these methods allowed fast, facile, and energy-efficient boron waste analysis,so as to determine valuable and harmful elements in waste streams as a prerequisite to evaluate their value as a secondary recourse.
